# Complications after radiofrequency ablation of hyperparathyroidism secondary to chronic kidney disease

**DOI:** 10.1080/0886022X.2023.2215334

**Published:** 2023-06-22

**Authors:** Li-Ping Lin, Miao Lin, Song-Song Wu, Wei-hua Liu, Li Zhang, Yi-ping Ruan, Mei-zhu Gao, Fu-Yuan Hong

**Affiliations:** a Department of Nephrology, Shengli Clinical College of Fujian Medical University, Fujian Provincial Hospital, Fuzhou, People’s Republic of China; b Department of Ultrasonography, Shengli Clinical College of Fujian Medical University, Fujian Provincial Hospital, Fuzhou, People’s Republic of China

**Keywords:** Chronic kidney diseases, secondary hyperparathyroidism, radiofrequency ablation, complications, recurrent laryngeal nerve injury, severe hypocalcemia

## Abstract

**Objective:**

To study the complications of ultrasound-guided radiofrequency ablation (RFA) in chronic kidney disease (CKD) patients undergoing renal replacement therapy with secondary hyperparathyroidism (SHPT).

**Methods:**

This retrospective study reviewed the clinical data, including general information, examination results, treatment times, time interval, and postoperative complications, of 103 SHPT patients who received ultrasound-guided RFA treatment from July 2017 to January 2021.

**Results:**

Of 103 patients, 52 required two sessions of RFA within a month. The incidence of recurrent laryngeal nerve injury at the second treatment was significantly higher than that at the first treatment (first session vs. second session, 5.77% vs. 21.15%; *p* = .021). Of all the enrolled 103 patients, 27 suffered complications after the first session of RFA. When we separated patients into complications group and non-complication group, we detected more ablated nodules in the complications group (*Z* = −2.222; *p* = .0026). Subgroup analysis further showed that the patients in the severe hypocalcemia group were younger (*p* = .005), had more ablated nodules (*p* = .003) and higher blood phosphorus (*p* = .012) and alkaline phosphatase (ALP) levels (*p* = .002). Univariate analysis showed that age, serum phosphorus, ALP, and number of ablated nodules were associated with a higher risk of severe hypocalcemia after the first session of RFA.

**Conclusions:**

An interval of more than 1 month between two treatments may help to avoid recurrent laryngeal nerve injury. Age, serum phosphorus, ALP, and number of ablated nodules were associated with a higher risk of severe hypocalcemia after the first session of RFA.

## Introduction

Secondary hyperparathyroidism (SHPT) is a common condition that affects patients with kidney failure. As the estimated glomerular filtration rate (eGFR) falls below 45 mL/min/1.73 m^2^ or less, abnormalities in calcium and phosphate levels stimulate an increase in parathyroid hormone (PTH) secretion [1]. SHPT has been linked with renal osteodystrophy, vascular calcification, muscle spasm, poor health-related quality of life, and increased mortality [2]. The National Kidney Foundation Working Group on Improving Global Renal Outcomes (KDIGO) indicated that SHPT screening and management should be initiated in all patients with stage 3 chronic kidney disease (CKD) (eGFR < 60 mL/min/1.73 m^2^) [3].

Early-stage SHPT can be effectively treated by phosphorus binders, vitamin D analogs, and calcimimetics [4]. However, these treatments do not always provide adequate control of SHPT, particularly at the late stage. Poor medication compliance, drug resistance, and drug side effects make treatment less effective and more expensive. Parathyroidectomy (PTX) should be considered in patients with severe SHPT [5]. PTX can improve hypercalcemia, hyperphosphatemia, and tissue calcification and reduce the risks of cardiovascular events and overall mortality in dialysis patients. However, SHPT patients with serious comorbidities are at increased risk of anesthesia-related morbidity and mortality, being deemed not fit for surgery. Thus, thermal ablation can be introduced as an alternative therapeutic option for these patients [6]. In addition, RFA causes fewer complications, such as nerve damage, bleeding, infection, and fever, and reduces the duration of hospitalization [6,7].

RFA has been shown to be particularly feasible for treating SHPT patients with CKD whose PTH level is >800 pg/mL [8]. RFA also presented multiple treatment advantages for SHPT patients with previous PTX [9]. Our previous studies demonstrated that RFA has similar clinical efficacy as surgery for treating refractory SHPT [10]. However, the sample sizes of these studies were small, and neither of these studies reported the safety of ultrasound-guided radiofrequency ablation (RFA) in treating SHPT. The goal of this study was to elucidate the risk factors of complications after RFA using a larger sample size.

## Materials and methods

### Patients

This study was approved by the Ethical and Scientific Review Board of Fujian Provincial Hospital (K2022-08-033). Written informed consent was obtained from all patients before they underwent RFA. We confirm that all methods were performed in accordance with the relevant guidelines and regulations. From July 2017 to January 2021, we retrospectively studied 108 patients who were diagnosed with SHPT caused by CKD. The inclusion criteria were as follows: (1) medically refractory SHPT (defined as PTH elevated >6 months despite maximal management with vitamin D analogs and calcimimetics); (2) PTH ≥ 800 ng/mL; and (3) at least one hyperplasic parathyroid nodule clearly >1 cm shown on US. The exclusion criteria were (1) primary hyperparathyroidism; (2) abnormal coagulation function; and (3) severe cardiopulmonary dysfunction.

### Pre-ablation examination and preparation

All patients’ demographics, primary disease, and dialysis history were recorded. The size and number of nodules, internal texture (solid or cystic), shape, echogenicity, margin, presence of calcification, and adjacent structures were carefully scrutinized by ultrasonography (USG). Blood tests assessing creatinine, alkaline phosphatase (ALP), calcium, phosphorus, PTH, hemoglobin, and ferritin were conducted before RFA.

### Key points of RFA techniques

**Figure 1.**
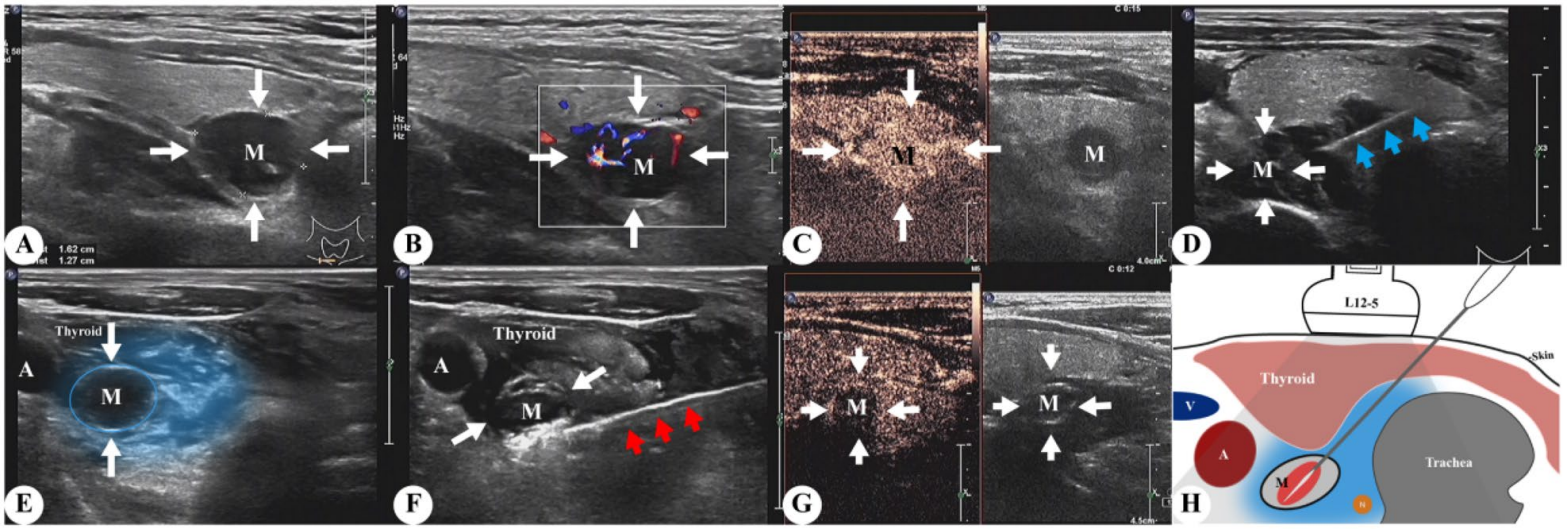
(A, B) B scan ultrasound and color Doppler imaging showing a right sided inferior parathyroid adenoma (white arrow, M), (C) image after injection of ultrasound contrast, (D) image demonstrating the position of the RFA needle (blue arrow), (E) layer of hypoechoic fluid along the adenoma (blue region) created by hydro-dissection, (F) image during RF ablation depicts parathyroid adenoma (M) and RFA needle (red arrow), (G) CEUS post-procedure demonstrating complete ablation, (H) schematic illustration depicting the procedure and its limits. RFA: radiofrequency ablation; CEUS: contrast-enhanced ultrasound.

RFA procedures were performed by the same team with several years of experience in interventional ultrasound. USG and contrast-enhanced ultrasound (CEUS) were performed using gray scale imaging with an iU22 USG scanner and an L12-5 high-frequency linear transducer. Thermal ablation of hyperplastic parathyroid glands was conducted using a radiofrequency generator (host mode VRS01, STARMed, Seoul, South Korea) and a disposable monopolar electrode (model 1807s07F; working electrode length 7 mm). Patients were placed in the supine position with the neck region fully exposed and sterilized. After local anesthesia with 2% lidocaine, a buffering zone was created by injection of 20 mL 0.9% sterile saline solution around the hyperplastic parathyroid to protect the adjacent recurrent laryngeal nerves and vessels from thermal injury. The RFA needle was inserted into the targeted glands, and hyperplastic parathyroid glands were ablated by moving the electrode step-by-step. USG and CEUS were performed again after ablation to confirm that the ablation was complete. Communication with the patient during RFA helped to detect any voice changes to prevent further damage. The ablation process of the RFA is illustrated in Figure 1.

### Complications

Complications included severe hypocalcemia, recurrent laryngeal nerve injury, and neck pain. Severe hypocalcemia was defined as a blood calcium level of less than or equal to 1.5 mmol/L, with or without numbness and convulsions in the limbs and face. Recurrent laryngeal nerve injury refers to unilateral or bilateral vocal cord paralysis shown by laryngoscopy or clinical manifestations of dysphagia, drinking cough, or hoarseness.

### Statistical analysis

Statistical analysis was performed using IBM Corp., released 2013, IBM SPSS Statistics for Windows, Version 22.0, IBM Corp. (Armonk, NY). Continuous variables are presented as the mean and standard deviation (SD) or median (interquartile range (IQR)) and were analyzed using the *t*-test (for normally distributed data) or Mann–Whitney’s *U*-test (for skewed data). Categorical variables are presented as frequencies and were analyzed using the Chi-squared or Fisher’s exact test. Multivariate logistical regression analysis was used to screen independent features for the occurrence of severe hypocalcemia. A *p* value < .05 was considered statistically significant.

## Results

### Baseline characteristics

A total of 103 maintenance dialysis patients who underwent RFA for severe forms of SHPT in our hospital from July 2017 to January 2021 were selected. Their baseline clinical characteristics are summarized in Table 1.

**Table 1.** Baseline clinical characteristics of patients (*n* = 103).

Characteristics	Data
Sex	
Male, *n* (%)	58 (56.3%)
Mean age (min–max)	50.8 ± 13 (19–75)
Renal replacement therapy	
Peritoneal dialysis	29
Peritoneal dialysis duration (years)	5.4 ± 3.4
Hemodialysis	74
Hemodialysis duration (years)	8.4 ± 4.3

The results are expressed as: number (*n*), percent (%), mean ± standard deviation (SD), and min–max.

### RFA

Complete ablation was achieved in all 103 patients, 41 in one session, 52 in two sessions, five in three sessions, three in four sessions, one in five sessions, and one in six sessions. Table 2 shows the serum biochemical data before and after RFA. PTH levels were significantly decreased 12 h after RFA (1339 (1262.77) pg/mL vs. 488.2 (859.92) pg/mL, *p* < .01). The mean calcium level decreased from 2.31 ± 0.19 mmol/L before RFA to 2.21 ± 0.02 mmol/L at 4 h and to 2.09 ± 0.22 mmol/L at 12 h after RFA (*p* < .05). The mean phosphate level also decreased from 2.24 ± 0.45 mmol/L before RFA to 2.01 ± 0.04 mmol/L at 4 h and to 1.98 ± 0.04 mmol/L at 12 h after RFA (*p* < .05).

**Table 2.** Comparison of differences in parameters before and after RFA.

	Parathyroid hormone (pg/mL)	Serum calcium (mmol/L)	Serum phosphorus (mmol/L)
Before RFA	1339 (915.23–2178)	2.31 ± 0.19	2.24 ± 0.45
4H post-RFA	–	2.21 ± 0.02	2.01 ± 0.04
12H post-RFA	488.2 (182.83–1042.75)	2.09 ± 0.22	1.98 ± 0.04

RFA: radiofrequency ablation.

### Complications

The main postoperative complications were severe hypocalcemia, recurrent laryngeal nerve injury, and neck pain. All recurrent laryngeal nerve injuries were resolved within 2–3 months, and all severe cases of hypocalcemia were rapidly resolved with calcium supplementation.

There were 52 patients who received the first two sessions of RFA within a month, of whom five patients (5/52, 9.62%) had complications after the first session of RFA, and 17 patients (17/52, 32.69%) had complications after the second session of RFA. The overall complication rate was similar (*p* > .05). Furthermore, complications were analyzed using subgroup analysis. The incidence of recurrent laryngeal nerve injury at the second treatment was significantly higher than that at the first treatment (first session vs. second session, 5.77% vs. 21.15%; *p* = .021). The incidence of severe hypocalcemia at the second treatment was 3.85% (2/52), but there was no significant difference when compared with that at the first treatment, which was 13.46% (7/52).

Of all the enrolled 103 patients, 27 suffered complications after the first session of RFA. When we separated patients into complications group and non-complication group, we detected more ablated nodules in the complications group (Table 3). There were three (2) nodules ablated in the complications group and two (1.8) nodules ablated in the noncomplications group (*Z* = −2.222; *p* = .0026). However, there was no difference in sex, age, dialysis duration, dialysis modality, albumin, blood creatinine, ALP, blood calcium, phosphorus, PTH, hemoglobin, ferritin, number of ablated nodules, or maximum size of nodules between the two groups.

**Table 3.** Comparison results of indicators between complication and non-complication groups.

Characteristics	Complication group, *n* = 27	Non-complication group, *n* = 76	*p*
Male/female, *n*	15/12	43/33	.927
Age, years, mean (SD)	49.04 (14.08)	51.25 (11.31)	.422
Hemodialysis/peritoneal dialysis, *n*	22/5	52/24	.195
Dialysis duration, years, mean (SD)	7.50 (4.27)	7.63 (4.26)	.885
Albumin, g/L, mean(SD)	33.95 (4.90)	34.37 (5.34)	.717
Serum creatinine, μmol/L, mean (SD)	953.15 (273.88)	935.40 (355.12)	.814
Alkaline phosphatase, U/L, *M* (IQR)	270 (711)	213.55 (259.8)	.151
Serum calcium, mmol/L, mean (SD)	2.38 (0.18)	2.39 (0.23)	.855
Serum phosphorus, mmol/L, mean (SD)	2.36 (0.47)	2.37 (0.58)	.934
Parathyroid hormone, pg/mL, *M* (IQR)	1623 (1897)	1500 (1452.3)	.349
Ferritin, μg/L, *M* (IQR)	314.3 (246.6)	241.05 (228.6)	.252
Hemoglobin, g/L (SD)	98.55 (18.76)	96.41 (22.83)	.662
Nodule maximum size, mm, mean (SD)	22.13 (19.90)	17.97 (5.90)	.294
Ablated nodule number, *n*, *M* (IQR)	3 (2)	2 (1.8)	.026

*M* (IQR): median (interquartile range).

Data are presented as mean (SD) or *M* (IQR).

Severe hypocalcemia occurred in 10 patients, recurrent laryngeal nerve injury occurred in 16 patients, and neck pain occurred in two patients, including one patient who suffered both severe hypocalcemia and recurrent laryngeal nerve injury. Subgroup analysis further showed that there were no significant differences between the laryngeal recurrent nerve injury group and the no laryngeal recurrent nerve injury group in any of the abovementioned variables (*p* > .05). Compared with the patients without severe hypocalcemia, the patients with severe hypocalcemia were younger (*p* = .005), had more ablated nodules (*p* = .003) and higher blood phosphorus (*p* = .012) and ALP levels (*p* = .002). High ALP remained significantly associated with severe hypocalcemia, whether considered normal distribution data (after log transform) or skewed data (Table 4). Univariate analysis showed that age, serum phosphorus, ALP, and number of ablated nodules were associated with a higher risk of hypocalcemia after the first session of RFA (Table 5).

**Table 4.** Comparison results of indicators between SH and no SH groups.

Characteristics	SH group, *n* = 10	No SH group, *n* = 93	*p*
Male/female, *n*	4/6	54/39	.326
Age, years, mean (SD)	40.70 ± 12.53	51.89 ± 11.54	.005
Hemodialysis/peritoneal	8/2	66/27	.721
*Dialysis, n*			
Dialysis duration, years, mean (SD)	7.95 ± 5.85	7.56 ± 4.08	.787
Albumin, g/L, mean (SD)	33.03 ± 4.91	34.39 ± 5.25	.433
Serum creatinine, μmol/L, mean (SD)	922.61 ± 234.83	941.93 ± 344.44	.863
Alkaline phosphatase, U/L, *M* (IQR)	652.5 (854.3)	194.2 (262.3)	.002
Serum calcium, mmol/L, mean (SD)	2.31 ± 0.15	2.40 ± 0.22	.208
Serum phosphorus, mmol/L, mean (SD)	2.61 ± 0.25	2.34 ± 0.57	.012
Parathyroid hormone, pg/mL, *M* (IQR)	2593 (2300.8)	1500 (1269.8)	.078
Ferritin, μg/L, *M* (IQR)	314.3 (173.9)	245.1 (238.1)	.406
Hemoglobin, g/L, mean (SD)	92.20 ± 21.81	97.48 ± 21.82	.468
Nodule maximum size, mm, mean (SD)	19.25 ± 4.61	17.91 ± 6.08	.501
Ablated nodule number, *n*, *M* (IQR)	4 (0.5)	2 (1.5)	.003

SH: severe hypocalcemia; *M* (IQR): median (interquartile range).

Data are presented as mean (SD) or *M* (IQR).

**Table 5.** Logistic regression analysis of risk factors of hypocalcemia.

Variables	OR	95%CI	*p*
Age, 1 year	0.861	0.764, 0.971	.015
Alkaline phosphatase, 1 U/L	1.003	1.001, 1.005	.009
Serum phosphorus, 1 mmol/L	117.753	2.244, 6177.866	.018
Ablated nodule number, per 1 nodule	7.969	2.009, 31.609	.003

## Discussion

In our previous study, Zhang et al. [10] demonstrated that US-guided RFA is an effective and minimally invasive treatment for SHPT patients and is not inferior to PTX in terms of safety and efficacy. In the current study, we used US-guided RFA to treat parathyroid hyperplasia in 103 patients with SHPT caused by CKD and showed that serum calcium, phosphorus, and PTH levels decreased significantly after RFA in patients with SHPT, which was consistent with the results of our previous study.

The main complications encountered in the present study included severe hypocalcemia, recurrent laryngeal nerve injury, and neck pain. A ‘liquid insulation layer’ method was adopted to protect the adjacent recurrent laryngeal nerves and vessels from the thermal injury. CEUS makes it easier to judge the size and location of the gland and reduces the incidence of complications [11]. SHPT patients usually undergo RFA treatments at two separate times to avoid bilateral recurrent laryngeal nerve injuries. According to the literature, hypocalcemia was more severe in patients who underwent a single session of RFA than in patients who underwent two sessions [12]. However, there has been no study evaluating the incidence of complications within the same individual at different sessions. Our study included 52 patients who underwent two sessions of RFA within a month and showed no difference in the incidence of severe hypocalcemia after the first or second treatment. However, the incidence of recurrent laryngeal nerve injury at the second treatment was significantly higher. The results suggest that an interval of more than 1 month between two treatments may help to avoid recurrent laryngeal nerve injury, and the appropriate interval should be further studied.

Hypocalcemia is a common complication after invasive treatment of SHPT [13,14]. Our study found that 22.1% of patients experienced severe hypocalcemia after the first session of RFA. The predisposing, independent risk factors contributing to its development were younger age, higher pre-RFA phosphorus, higher pre-RFA ALP levels, and a greater number of ablated glands.

The influence of age on postoperative calcium concentration was uncertain. In the present study, both the single-factor and multiple-factor logistic regression analyses identified age as an independent risk factor for postoperative hypocalcemia. The rapid decline in circulating PTH following ablation triggers reduced bone resorption and increased bone formation. As younger individuals have a higher osteogenic ability, they are more likely to develop severe hypocalcemia [13]. High serum phosphorus was defined as a risk factor for severe hypocalcemia after microwave ablation, which was in accordance with previous studies. This can be mainly explained by hungry bone syndrome [15,16].

The preoperative ALP level has been reported as an independent risk factor for the prediction of severe hypocalcemia in previous studies based on PTX [17,18]. Ren et al. [19] demonstrated that the preoperative ALP concentration was a risk factor for hypocalcemia after invasive treatment of SHPT. We found that the postoperative low calcium level in the patients was significantly correlated with a higher pre-RFA ALP level. ALP is an extracellular enzyme of osteoblasts and is a biomarker of osteoblast maturity and bone turnover [20]. Therefore, the higher the ALP, the higher the bone transport state, and thus the higher the incidence of hypocalcemia after RFA.

According to the present results, the number of glands ablated was indeed proven to be an independent risk factor for post-RFA severe hypocalcemia. The results of this study were consistent with those of a previous retrospective cross-sectional study based on microwave ablation [16]. Therefore, for patients with multiple ablated glands, ablations can be performed several times to reduce post-RFA severe hypocalcemia on the basis of effective treatment.

This study had some limitations. First, as a retrospective study, there may have been selection bias, and thus, additional prospective studies are needed to establish more definitive results. Second, the number of patients enrolled was relatively small, and long-term studies with larger samples are needed to explore the efficacy and safety. Third, a comparative study between RFA and surgery is still necessary.

## Conclusions

The obtained findings confirm that an interval of more than 1 month between two treatments may help to avoid recurrent laryngeal nerve injury, and the appropriate interval is the next to be studied. We also demonstrated that age, serum phosphorus, ALP, and number of ablated nodules were associated with a higher risk of severe hypocalcemia after the first session of RFA.

## Data Availability

Datasets are available from the corresponding author on reasonable request.
